# Status of *Pratylenchus coffeae* in banana-growing areas of Tanzania^☆^

**DOI:** 10.1016/j.pmpp.2018.08.002

**Published:** 2019-01

**Authors:** Nessie D. Luambano, Beatrice E. Kashando, Minza M. Masunga, Ambilikile E. Mwenisongole, Magreth F. Mziray, Jeremiah E. Mbaga, Renifrida M. Polini, Doreen M. Mgonja

**Affiliations:** a Sugarcane Research Institute-Kibaha, P. O. Box 30031, Kibaha Coast, Tanzania; b Mikocheni Agricultural Research Institute, P. O. Box 6226, Dar es Salaam, Tanzania

**Keywords:** Banana, *Musa*, Plantain, Root-lesion nematodes

## Abstract

*Pratylenchus coffeae* is among the plant parasitic nematodes contributing to yield losses of banana. To determine the status of *P. coffeae*, a survey was conducted in banana-growing regions of Tanzania and samples collected. The results indicated that in 2015 there was an increase in total counts of *P. coffeae* extracted from roots compared to that reported in 1999 in Unguja West, North and South. Moreover, we noted its presence for the first time in mainland Tanzania. Generally, the densities of *P. coffeae* were high on banana roots collected at 500–1000 m above sea level. This information on the status of *P. coffeae* is important in planning management of nematodes in Tanzania.

## Highlights

• *Pratylenchus coffeae* occurred in about 80% of ten regions where banana was surveyed.
• The incidences of *P. coffeae* were above 50% for Unguja North, Mbeya and Ruvuma.
• The population densities of *P. coffeae* increased from 74 to 2996/100 g of fresh roots in Unguja island.
• In mainland Tanzania, *P. coffeae* was reported for the first time.
• High population densities of *P. coffeae* occurred on banana roots grown at 500–1000 m a.s.l.

## 1 Introduction

Banana (*Musa* spp.) is one of the most important food crops in the Great Lakes region of Africa (including Burundi, Congo, Kenya, Rwanda, Uganda and Tanzania) and this region has the greatest level of banana consumption worldwide [1]. Banana is grown and consumed all over Tanzania, and the importance of production locally varies depending on the importance of the crop to the specific area. For example, banana is grown as staple food in Kagera and Kilimanjaro regions in a coffee–banana field system and hence is widely grown and consumed [2,3]; however, in areas like Mbeya, most of the bananas grown are sold in the cities of Mbeya and Dar es Salaam [4]. Tanzania is divided into two parts: mainland Tanzania and the islands of Zanzibar in the Indian Ocean. Banana production is high in the cool highland areas of the mainland such as Kagera, Arusha, Kilimanjaro and Mbeya where banana is staple food and the main source of daily consumed carbohydrate. However, most of the areas in the Zanzibar islands of Unguja and Pemba grow banana and plantain in small gardens for the purpose of producing fried snacks. Those who grow banana as the main staple food, especially in mainland Tanzania, sell the surplus for cash in nearby towns and cities or process the crop into banana beer or wine [5]. Rapid growth of urban and informal towns, especially in the mainland, and changes in food behaviour may increase demand for banana as food and fruit [6,7]. Moreover, improvement to technology and value addition of banana products such as banana biscuits, flours, bread, doughnuts and wine [8,9] increase demand for improved production. According to FAOSTAT [10], average annual banana productivity during 2005–2014 was less than 7 t/ha. However, banana has the potential to produce 30 t/ha, which could be achieved in Tanzania with improved management practices [11].

Productivity of bananas in the Great Lakes region of Africa has greatly declined since the 1970s, and is now 7–42% of its potential [12]. Some of the reasons for reduced banana yields are poor soil fertility and pests and diseases [13]. In particular, plant parasitic nematodes are extremely damaging, causing yield losses of more than 40% across all banana crops in Africa [14], and 20% worldwide [15,16].

The main nematode species known to affect banana crops worldwide are *Pratylenchus goodeyi*, *Radopholus similis* and *P. coffeae.* Of these, *P. goodeyi* and *R. similis* have been previously reported in Tanzania [17,18]. The former is thought to be indigenous to Tanzania but the latter is introduced and confined to the humid lowlands [19]. However, the current situation of these species in Tanzania in relation to localisation and level of pathogenicity is unknown. This is mainly a result of different challenges, some of which were reviewed by De Waele and Elsen [20], which includes lack of adequately equipped nematology laboratories (due to the microscopic nature of nematodes), trained taxonomists, routine nematode monitoring surveys and financial support. Generally, little research has been conducted on banana nematodes in Tanzania and thus there is scant information on the status of some nematodes.

*Pratylenchus coffeae* (Zimmermann) Filpjev and Schuurmans-Stekhoven is one of the few *Pratylenchus* species known to be a pathogen of banana, and causes root lesions [21]. It is a widespread pest that causes serious damage to banana plants in Latin America, but has not been previously documented in mainland Tanzania. This may be due to both the lack of resources to collect information throughout the major banana-growing areas and its absence at that time from the few surveyed areas [18]. However, *P. coffeae* was first reported by Rajab et al. [22] in the Zanzibar islands, a small isolated part of Tanzania. The roots samples collected from all regions of Unguja were extracted to get nematodes where the population density of *P. coffeae* was 74/100 g of fresh roots [22]. Apart from banana, *P. coffeae* has been known to cause quantity and quality losses to food, cash crops and spices. In Japan, *P. coffeae* can cause serious losses of sweetpotato [23], the crop which ranks fourth in importance among food crops in Tanzania and is grown in all areas where banana is grown [24]. Coffee is a good host for *P. coffeae* and is the leading cash crop in Kagera, Kilimanjaro and part of Ruvuma in mainland Tanzania, areas where they also grow banana for staple food and sometimes intercropped with coffee [25]. The spices ginger and turmeric are susceptible to *P. coffeae* [26] and play major roles in the economy of Zanzibar. Thus, increases in *P. coffeae* could directly affect other important crops in the country.

The presence and distribution of different nematodes in the area varies with time and is likely due to movement of plant material through the common practice of exchange between farmers or introduction from one country to another [27]. Banana is vegetatively propagated and thus farmers collect materials/corms from neighbours or bordering countries and this can introduce new nematode species [[28], [29], [30]]. Therefore, information on the specific type and abundance of nematodes is required to assess potential nematode damage in any new banana production area.

This study was conducted to assess the status of *P. coffeae* in the banana production systems of Zanzibar and mainland Tanzania. Information on status of *P. coffeae* will be useful for development of strategic nematode management and improving banana production in Tanzania.

## 2 Materials and methods

### 2.1 Study area and sampling

**Fig. 1:**
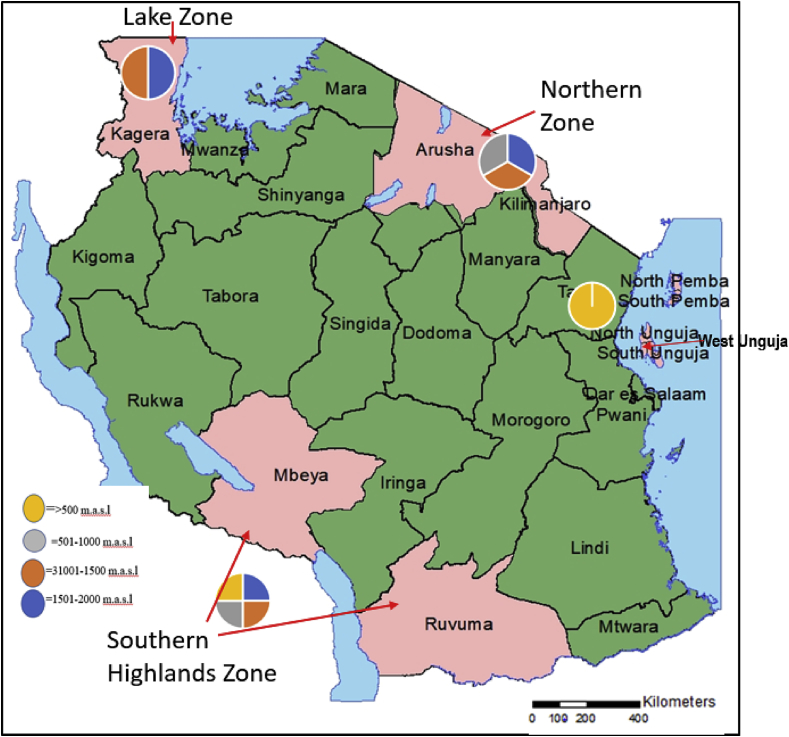
Map showing regions in Tanzania where samples for the survey of banana nematodes were collected (pink colour); pie charts show proportions of different altitudes for each agro-ecological zone surveyed.

The study was conducted in 10 major banana-growing areas across three agro-ecological zones of mainland Tanzania and one from Zanzibar (Fig. 1). The zones and regions for mainland Tanzania follow: the Lake Zone (Kagera region), the Southern Highlands Zone (Mbeya and Ruvuma regions) and the Northern Zone (Arusha and Kilimanjaro regions). The five regions in Zanzibar were North Pemba, South Pemba, North Unguja, South Unguja and West Unguja. The survey time for one agro-ecological zone was about five days but this was in different months of 2015 for each zone. The month chosen for each zone was according to availability of moisture in soil, which attracts nematodes to the rhizosphere from where soil and root samples were collected. The agro-ecological zones differ in terms of weather conditions including the rainy season and temperature. The maximum and minimum temperatures for three months prior to sampling for each zone are shown in Table 1.

**Table 1:** Data on maximum and minimum temperature (°C) for the three months before the month of sample collection. All data were collected during 2015 except for December which was in 2014.

Zone	Lake			Northern			Southern			Zanzibar		
Month	Dec	Jan	Feb	May	June	July	Nov	Dec	Jan	Feb	Mar	April
High	28.4	27.9	27.9	23.3	22.9	23.2	26.5	25.1	24.7	34	32.5	31.5
Low	18.5	18.4	18.8	16.3	14	13.9	14.6	115.0	14.8	25.1	25	25.21

In this survey of the 10 regions, we randomly collected 314 composite samples of each of soil and roots to make a total of 628. The samples were collected following a previously described procedure [16]. A 20 × 20 × 20 cm hole was dug adjacent to the corm of the banana plant, and banana roots and soil were collected and placed in labelled plastic bags. From each field, samples were taken from five plants selected at random and pooled to form composite root and soil samples. From the composite sample, about 500 g of soil and 10 roots (length 10–15 cm) were packed into labelled plastic bags and stored in cool boxes ready for transfer to the nematology laboratory at the Sugarcane Research Institute, Kibaha, Tanzania. Data on soil pH, texture, nitrogen, phosphorus and potassium for the regions surveyed were collected, based on previous research and a database, for comparison with nematode data (Table 2). Geographical location data (altitude, latitude and longitude) were also collected from the surveyed areas.

**Table 2:** Soil properties for 10 regions where banana samples were surveyed as cited from different published reports.

Region	pH (water)	Texture for 0–20 cm depth (%)			N (%)	P (mg/kg)	K (cmol (+)/kg)	Source
		Sandy	Silt	Clay				
Kagera	4.5–6.7	48	30	22	0.04–0.26	20–25	0.00–1.92	ARI-Mlingano
Kilimanjaro	4.8–9.8	58	24	18	0.21–13.7	4.86	0.72	ARI-Mlingano
Arusha	5.8–8.2	10	15	75	0.21–13.8	4.86	0.72	[31]
Ruvuma	5.4–6.4	68	12	20	0.1–0.3	0.20–3.64	0.6–1.2	ARI-Mlingano
Mbeya	4.4–6.6	44	31	25	0.1–0.3	0.20–3.64	0.6–1.2	[32]
N. Unguja	6.8	25	43	32	0.25	3.24	0.09	ARI-Kizimbani
S. Unguja	7.2	36	38	26	0.17	1.8	0.03	ARI-Kizimbani
N. Pemba	5.4	71	12	17	0.13	6.8	0.05	ARI-Kizimbani
S. Pemba	6.3	35	20	45	0.18	3.28	0.09	ARI-Kizimbani
W. Unguja	7.6	27	53	20	0.31	4.1	0.03	ARI-Kizimbani

**Note**: N. Unguja = North Unguja, S. Unguja = South Unguja, N. Pemba = North Pemba, S. Pemba = South Pemba, W. Unguja = West Unguja, N = nitrogen, P = phosphorus and K = potassium where cmol(+)/kg is centimoles of positive charge per kilogram of soil. ARI-Mlingano is Central Soil Laboratory, Agricultural Research Institute, Mlingano, Tanzania, and ARI-Kizimbani is Agricultural Research Institute, Kizimbani, Zanzibar.

### 2.2 Nematode identification, counting and incidence

**Fig. 2:**
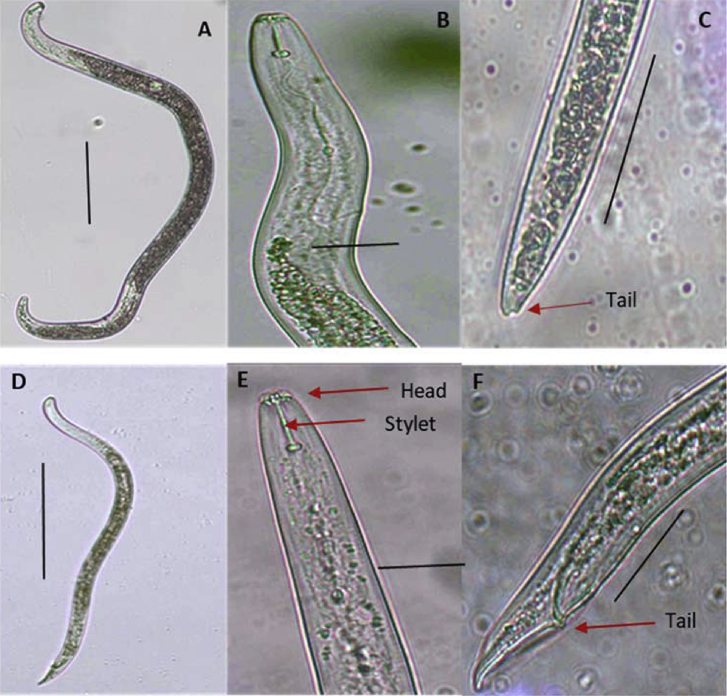
*Pratylenchus coffeae* photographed using a compound microscope (×40) in the laboratory at the Sugarcane Research Institute-Kibaha, Tanzania. A: Female length; B: female anterior region with the head at the upper end and full body width at the bottom; C: truncated female tail tip indicated by red arrow; D: male length; E: male anterior region with head at the upper end and stylet indicated by red arrow and full body width at the bottom; and F: male tail with spicule indicated by red arrow. Scale bars (μm): A = 570.6, B = 16.7, C = 31.6, D = 471.1, E = 11.9 and F = 37.3.

Nematodes from soil and roots were extracted by the modified Baermann technique using 100 mL of soil and 5 g of roots as described by Hooper et al. [33]. Soil and macerated roots were incubated for 48 and 24 h, respectively. Microscopy was used for morphological identification of structures on *P. coffeae* (Fig. 2) that distinguished it from other *Pratylenchus* species. The morphological features and specific details for *P. coffeae* used were compared to the information provided by Castillo et al. [34]. Specific features illustrated for *P. coffeae* are presence of two annuli on the labial region, round to oblong shape of stylet basal knobs and truncated or hemispherical tail shape [34]. Nematode extracts were counted using a 2-mL aliquot on a counting slide (designed by Sikora and made at Bonn University, Germany) under a Leica 2500 (Leica Microsystems CMS GmbH, Wetzler, Germany) compound microscope at ×20 magnification. Using the same microscope, the nematodes were clearly identified with support of immersion oil at ×100 magnification and photos were captured at ×40 magnification.

Nematode abundance was obtained from the average of three nematode counts according to Bezooijen [35] using the following formula:

$$Nematode\;abundance=\frac{Nematode\;counts\;from\;three\;samples\;\left(1+2+3\ldots \right)}{Total\;number\;of\;counts}$$

Also, percentage incidences were calculated using the total number of fields found with nematodes compared with the total number of fields surveyed, by adopting the formula of Esfahani and Nasr [36] as follows:

$$\%\;incidence=\frac{Number\;of\;samples\;with\;nematodes}{Total\;number\;of\;samples\;collected}\times 100$$

### 2.3 Molecular identification

PCR was conducted to confirm the morphological identification. Twenty nematodes from each agro-ecological zone were used for amplification of the ITS and 28S regions of the rDNA.

### 2.4 DNA extraction from nematodes

The DNA extraction was conducted according to protocol illustrated by Harris et al. [37] with few modifications. One or two nematodes from the same sample were handpicked and placed on a glass slide with 10 μL of extraction buffer (1 M Tris-HCl, pH 8.0 and 2.5 mM MgCl). The mixture was ground and 40 μL of extraction buffer added to make 50 μL of solution. The solution was transferred into a 1.5-mL Eppendorf tube; 1% sodium dodecyl sulphate and 0.5 μL of proteinase K (New England Biolabs, Hitchin, UK) were added, and the solution incubated at 65 °C for 30 min.

The lysate was then extracted with an equal volume of phenol/chloroform/isoamyl at the ratio 25:24:1. The solution was mixed well and centrifuged at 4 °C for 5 min at 20,000 × *g* relative centrifugal force (RCF). The upper aqueous layer (50 μL) was transferred into a new tube, then 5 μL of cold 3 M NaAc (pH 5.2) and 2× volume of 98% cold ethanol was added, and then samples were precipitated at −20 °C for 1 h. The mixture was centrifuged at 4 °C for 15 min at 20,000 × *g* RCF. The DNA pellets were washed with cold 75% ethanol and re-suspended in 10 μL of sterilised double distilled water, then stored at −20 °C for further analysis.

### 2.5 DNA amplification

The extracted nematode DNA was used as a template for PCR amplification. The PCR was performed using two primer pairs which were designed by authors at Mikocheni Agricultural Research Institute: PC7ITSF (GAGCAGTCGTATTCGTCCGT) and PC7ITSR (AAGTTCAGCGGGTATTCACGTC) that amplifies the ITS1, 5.8S and ITS2 regions; and PC11LSUF (ACAAGTACCGTGAGGGAAAGTTG) and PC11LSUR (TCGGAAGGAACCAGCTACTA) that amplifies the 28S of rDNA. These primers are specific to *Pratylenchus* spp. The primers were designed from the internal transcribed spacer (ITS)1 and 2 and the 28 subunit (28S) of the ribosomal DNA (rDNA) region of nematodes using the primer design tool of the National Centre for Biotechnology Information (NCBI) (https://www.ncbi.nlm.nih.gov/tools/primer-blast/) as described by Ye et al. [38]. The PCR conditions were denatured at 94 °C for 3 min followed by 35 cycles of denaturation at 94 °C with 30 s, annealing at 61 °C for 45 s and extension at 72 °C for 2 min. A final extension was performed at 72 °C for 10 min. The amplicons were analysed on 1% agarose gel electrophoresis, photographed over a *trans*-illuminator and cleaned by ExoSap-IT (Affymetrix Inc., Santa Clara, CA, USA). The cleaned PCR products were added with their respective sequencing primer pairs and sequenced directly by Bioneer Corp, South Korea.

#### 2.5.1 Sequence analysis and phylogeny

The obtained molecular sequences were compared with other nematode species sequences available in GenBank through NCBI BLASTN homology search. The DNA sequences were analysed by molecular evolutionary genetics analysis (MEGA7) version 7.0 software [39]. The sequences were edited on Bioedit software ver. 7.0 (Carlsbad, CA, USA) and aligned by multiple sequence comparison by log-expectation (MUSCLE) in MEGA 7 software. Phylogenetic trees based on internal transcribed spacers (ITS1 and ITS2 linked by 5.8S) and 28S rDNA sequences were constructed using the maximum parsimony method.

### 2.6 Statistical analysis

All data on nematode counts were subjected to analysis of variance using the GenStat statistical package (14th edition, VSN International Ltd, Hemel Hempstead, UK). If required, the density of nematodes was square-root transformed. The means were compared using least significant difference at *P* < 0.05.

## 3 Results

**Fig. 3:**
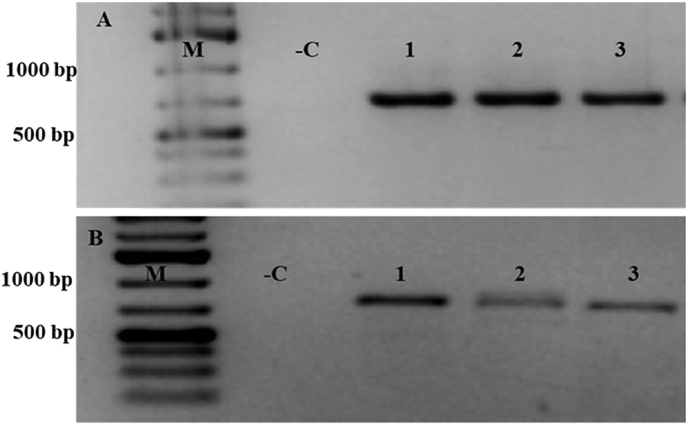
Gel electrophoresis for *Pratylenchus coffeae* obtained from banana roots collected from different agro-ecological zones of Tanzania. A and B represent *P. coffeae* amplified by primer pairs PC7ITSF/PC7ITSR (estimated size ∼676 bp) and PC11LSUF/PC11LSUR (estimated size ∼781 bp), respectively. Number **1**: Representative nematode sample isolated from Zanzibar, **2:** Southern Highlands Zone and **3:** Northern Zone. -C represents a non-template negative control and M is a DNA marker (1 kb plus).

**Fig. 4:**
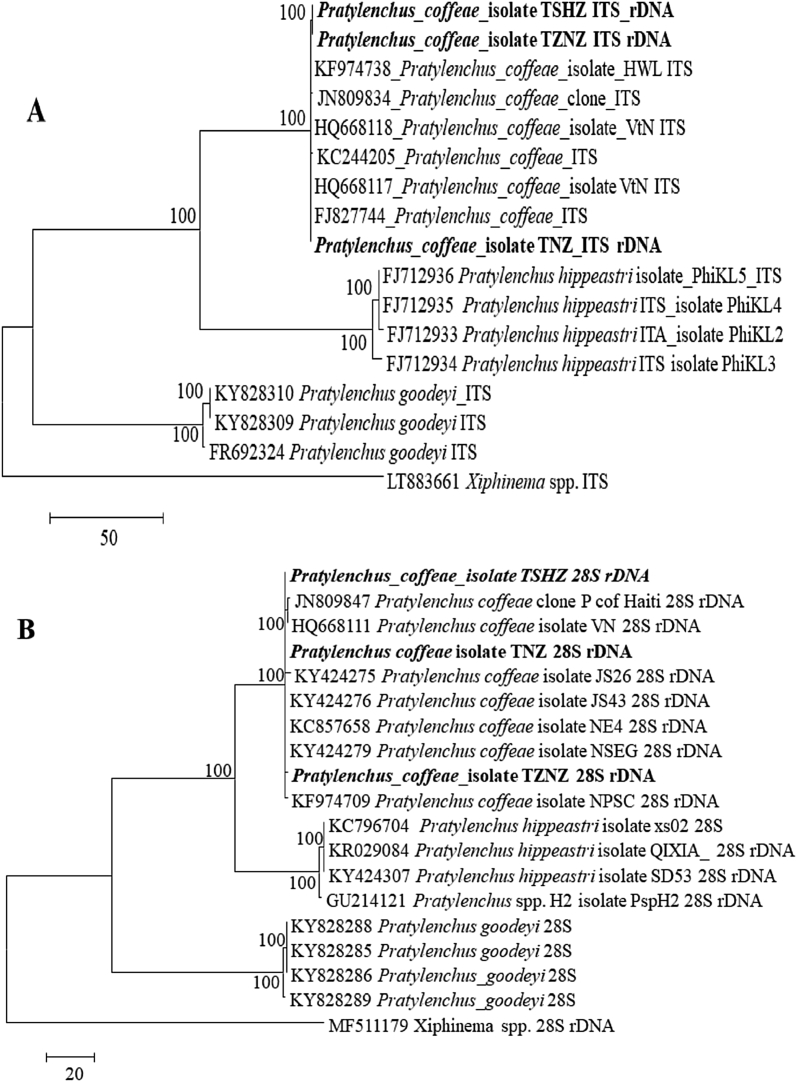
Phylogenetic relationships of *Pratylenchus coffeae* populations with other *Pratylenchus* spp. as inferred from maximum parsimony analysis of ITS (1 and 2) linked by 5.8S **(A)** and 28S **(B)** gene sequences of rDNA region.

Morphology of *P. coffeae* found in samples was confirmed using the tail shape of female *P. coffeae*, which was truncated (Fig. 2). Moreover, we also observed body annulation lying from side to side as described by Castillo et al. [34]. We also took measurements of some key parameters specific for *P. coffeae*: for females, length (430.3–516.0 ± 140.3 μm compared to standard of 370–690 μm) and vulva (%) (67.7–80.4 ± 22.4 compared to standard of 76–83); and for males, length (353.8–407.1 ± 64.0.0 μm compared to standard of 450–700 μm) and testis length (113.9–162.1 ± 79.5 μm compared to 278.9 μm). The PCR amplification using primer pairs PC7ITSF/PC7ITSR and PC11LSUF/PC11LSUR showed the correct fragment sizes for all nematode samples. Molecular characterisation confirmed the morphological and morphometric characterisation of *P. coffeae* nematodes through PCR (Fig. 3) and sequencing. The sequencing and phylogenetic relationship of the ITS and 28S rDNA sequences of *P. coffeae* populations identified in this study were clustered together with other *P. coffeae* sequences from GenBank and were more closely related compared to other *Pratylenchus* spp. (Fig. 4).

**Fig. 5:**
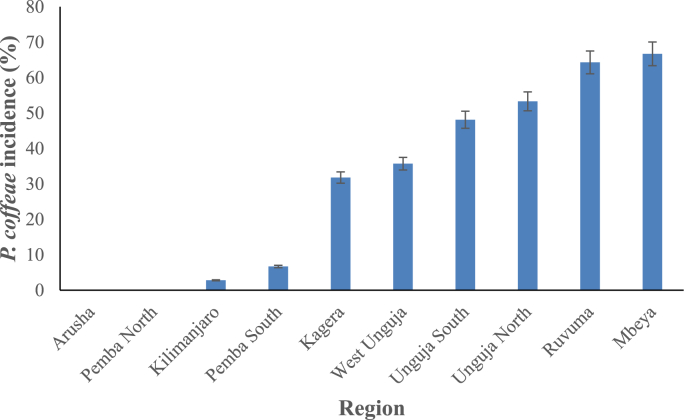
Percentage incidence of *Pratylenchus coffeae* from all banana root samples collected from 10 regions of Tanzania. The error bars represent a 5% error.

The incidence of *P. coffeae* in root samples was highest in Mbeya and Ruvuma regions followed by North Unguja and South Unguja (Fig. 5). The three regions of Unguja showed high incidence of *P. coffeae*, whereas there was low incidence in Kilimanjaro and Arusha. In addition, the range in incidence between regions was wide: 0% in Arusha to 70% in Mbeya. Of the regions, 30% had more than 50% incidence.

Nematode counts in the soil samples were high in the three regions of Unguja (Table 3). The highest counts were from North Unguja, followed by South Unguja and West Unguja. In contrast, nothing was found in Arusha, Kilimanjaro, Pemba South and Pemba North. The density of nematodes in root samples was highest in Mbeya and Ruvuma but these values did not significantly differ to those from South Unguja (*P* > 0.05; Table 3). The lowest nematode counts in root samples were from Arusha and North Pemba (Table 2).

**Table 3:** Distribution of *Pratylenchus coffeae* on soil and roots of banana in different regions of Tanzania.

Region	Soil	Root
Arusha	0.0 (0.0)	0.0 (0.0)
Kagera	192 (11.3)	1050 (32.2)
Kilimanjaro	0.0 (0.0)	42 (3.7)
Mbeya	425 (20.3)	1646 (50.9)
West Unguja	2161 (46.5)	809 (28.3)
North Pemba	0.0 (0.0)	0.0 (0.0)
South Pemba	0.0 (0.0)	25 (3.5)
Ruvuma	55 (6)	2780 (48.4)
North Unguja	3455 (57.2)	713 (26.5)
South Unguja	2275 (33.7)	1474 (38.4)
***P* (0.05)**	**0.004**	<**0.001**
**LSD (*P*** < **0.05)**	**24.37**	**18.21**

Values in brackets are square-root transformed means used in comparison with LSD (*P* < 0.05).

The highest nematode infections were for medium altitudes of 500–1000 m above sea level (m.a.s.l) (Table 4); in comparison there were significantly (*P* < 0.05) fewer roots infected with nematodes at elevations below 500 and above 1000 m a.s.l. Abundance of *P. coffeae* was highest in soil of elevations 0–500 m a.s.l. and lowest above 500 m a.s.l. (Table 4).

**Table 4:** *Pratylenchus coffeae* on banana roots and soil collected from different altitudes in Tanzania.

Region	Altitude zone	Root nematodes/100 g	Soil nematodes/100 g
Arusha	3	0	0
Arusha	4	0	0
Kagera	3	710	0
Kagera	4	1220	287.5
Kilimanjaro	3	0	0
Kilimanjaro	4	63	0
Mbeya	2	3118	512.5
Mbeya	3	1705	250
West Unguja	1	402	2165
North Pemba	1	0	0
South Pemba	1	27	0
Ruvuma	3	3725	82.5
Ruvuma	4	890	0
North Unguja	1	708	2970
South Unguja	1	1450	2350
(*P* < 0.05)		0.006	0.025

**Note**: Altitudes were grouped as 1 (0–500 m.a.s.l.), 2 (501–1000 m.a.s.l.), 3 (1001–1500 m.a.s.l.) and 4 (1501–2000 m.a.s.l.). Nematode densities are means from analysis of variance at *P* < 0.05.

## 4 Discussion

The survey results indicated the presence of *P. coffeae* in most regions of mainland Tanzania. The morphology and molecular identification confirmed its presence. The results on sequence and phylogenetic analysis demonstrated high relationship of these nematodes to other *Pratylenchus coffeae* from the database. Moreover, the data show that *P. coffeae* was spreading in most banana-growing areas in the Zanzibar islands of Unguja and Pemba. The nematodes survive in the warm areas of these islands, where air temperatures can reach 38 °C [40], and in the cooler areas of Mbeya with maximum temperatures of around 23 °C [41]. Information on temperature conditions (Table 1), especially for when the samples were collected, is important because previous reports indicated the influence of temperature on abundance of some nematode species such as *P. goodeyi* [17,42,43]. However, the results for *P. coffeae* collected from different altitudes and temperature showed no significant effect of temperature on abundance of *P. coffeae*. This is supported by results by Radewald et al. [44], who found maximum reproduction of *P. coffeae* at 29 °C but infection occurring at a range of 4–32 °C. In addition to temperature, previous data indicate that soil pH, rainfall and humidity affect the nematode population [45,46], which may have contributed to the distribution of *P. coffeae* in Tanzania. The effect of altitude on nematode populations was considered due to its relationship to temperature, because altitude is usually negatively correlated with temperature [47]. Although we did not find a direct relationship between temperature and nematode abundance, this nematode survived in medium to lower altitudes where temperatures are usually warm. These warm conditions might be one factor enhancing the population, by allowing a shorter life-cycle compared with cool conditions at high altitudes. Previous surveys in Tanzania for banana nematodes reported *P. coffeae* only in Zanzibar [22]. The current survey showed that *P. coffeae* was in either roots or soils of the banana samples from surveyed regions with the exception of two regions (Arusha and Pemba North), implying that one source of its invasion of the mainland may be the Zanzibar islands through movement of plant material. For the past two decades, *P. coffeae* has been newly reported across banana-growing areas in Africa. For example, the presence of *P. coffeae* was first reported on banana in Uganda after a nematode survey conducted in 1993 [17]. Moreover, *P. coffeae* was reported to cause 24% losses in bunch weight and the toppling of banana plants [48]. Similarly, a study conducted in Ghana showed that *P. coffeae* caused severe symptoms of dead roots, root necrosis and sucker corm lesions in bananas [49]. Together, these studies indicate that the presence of this nematode species on banana contributes to production losses. The results of our study are an alert to banana stakeholders to take such actions as developing effective management methods, sending awareness messages to banana growers and strengthening skills of crop inspectors before the problem in Tanzania becomes serious.

The current study revealed the presence of *P. coffeae* in about 80% of the samples collected, but there are no research data on the effect of this nematode on banana production. The results indicate a high chance of this nematode species surviving in a wide range of conditions where banana is grown if no effort is made to control movement of plant materials and increase farmer awareness. Traditionally, farmers source new vegetative materials from existing fields and neighbours [50]. In areas such as Unguja, where banana is left without recommended agronomic management and in which all studied soils (from three regions) contained *P. coffeae*, strategic management measures should be considered that must include cultural practices such as thinning of banana mats, sanitation and hot water treatment of planting material. When assessing the banana fields and collecting samples in Zanzibar, we noted that most banana mats contained 4–20 plants and were left to increase after planting the first crop – this high plant density might be one reason for high nematode incidences. The number of other crops intercropped was high, we found mixtures of taro, sweetpotato, cassava, eggplant, pumpkin, coconut, and orange cardamom and weed control was poor. Weeds such as Bermuda grass, nut grass and black nightshade are also host to different nematodes and can support them even when the crop is not in the field [51]. The danger of *P. coffeae* to banana production has been reported by a number of researchers [[52], [53], [54]] and the considerable damage to banana roots by *P. coffeae* has been reviewed in detail [20]. However, compared to the density of nematodes reported by Rajab et al. [22], in our survey the density had increased in Unguja regions and decreased in Pemba. These two islands (Unguja and Pemba) are isolated by water and the elapsed time of about two decades may have caused some differences in crops grown, microbial communities and farming activities. In addition, the nature of soil can enhance abundance and adaptability of *P. coffeae*.

This study indicates that incidence and density of nematodes were high in North and South Unguja and no nematodes were found in Arusha. The variation in nematode abundance can be caused by the nature of varieties grown in the two areas. Traditionally, Arusha has extensively grown East African highland banana which is an unique cooking diploid variety called mchare [55], whereas Unguja is dominated by variation of triploid varieties which include mzuzu, sukari ndizi and mkono wa tembo [22,56]. Moreover, according to the soil properties of the surveyed regions (Table 2), the soils in North and South Unguja were slightly acidic, loamy/slightly silty and of low nitrogen, whereas, that of Arusha was alkaline, clay and of high nitrogen content. The soil condition in Arusha might have hindered survival of *P. coffeae* in this soil and hence limited its availability. Soil properties and their relationship to incidence and density of *P. coffeae* imply that agronomic practices necessary for improvement of soil properties may significantly affect management of this nematode. The effects of clay texture and high soil nitrogen in hindering and reducing nematodes have been reported in different studies [8,57,58]. The information of our survey suggests that nematologists and other banana stakeholders should work together to develop effective measures such as cultural practices, biological agents and rotations to tackle *P. coffeae* to aid small-scale banana growers in Tanzania.

## 5 Conclusion

This study indicated that *P. coffeae* was widespread in banana-growing areas of Tanzania, from the mainland to the Zanzibar islands, regardless of climatic conditions. The results showed that important areas were infested; and further research should be concentrated in these regions to minimise the problem. Based on the survey results, we suggest pathogenicity should be studied in Tanzania to improve understanding of the relationship between *P. coffeae* and banana and the level of effects caused by this nematode. A knowledge and awareness programme is needed to help farmers apply available nematode management practices for improved banana production.

## Appendix A Supplementary data

Data Profile Data Profile
